# Cross‐sectional survey of the replacement of the Japanese term for dementia: Did it reduce discomfort in family members?

**DOI:** 10.1002/brb3.2012

**Published:** 2020-12-21

**Authors:** Katsuo Yamanaka, Naoya Todo, Mutsumi Yoshizawa, Tatsuji Uchida

**Affiliations:** ^1^ Faculty of Human Sciences University of Tsukuba Tsukuba Japan; ^2^ Course of Sign Language Interpretation College of National Rehabilitation Center for Persons with Disabilities Tokorozawa Japan; ^3^ Faculty of Health Science University of Tokyo Health Sciences Tokyo Japan

**Keywords:** dementia, family, stigma, structural equation modeling, terminology

## Abstract

**Objectives:**

Decreasing discrimination and stigma of dementia is an international issue. In 2004, the Japanese government changed the previous Japanese stigmatic term of dementia (“Chiho”) to the present one (“Ninchi‐sho”) a meaning near “neurocognitive disorder.” This study aimed to examine cross‐sectionally if the present term functioned well or not from the viewpoint of families of people with dementia (PWD), and to discover variables influencing their feelings of the term: the feelings about people surrounding PWD, and the family members’ and PWD’s attributes.

**Methods:**

Questions regarding the feelings about the present Japanese term and people surrounding PWD were asked to 155 family members accompanying PWD who visited three hospitals. For analyses, the degree of the discomfort about the present Japanese term was shown descriptively. The relationship of constructs of the feelings extracted by exploratory factor analysis (EFA) and the attributes was analyzed using structural equation modeling (*SEM*).

**Results:**

71.6% agreed that the present term discomforted them less than the previous one. Only 13.2% thought that the present term was discriminatory. However, about one third of the participants felt discomfort when they used even the present term. Using the constructs extracted by EFA, the analysis of *SEM* revealed that the negative feelings of the terminology were affected by hesitation to disclose to surrounding people that their family member had dementia, which the attributes of younger family members, wives, husbands, and siblings influenced. Moreover, because of disclosing the dementia, the feelings of support from people alleviated the feelings of hesitation, influenced by sex (female).

**Conclusions:**

It was suggested that overall, the present term successfully reduced discomfort in families, compared with the result of the previous term surveyed by the Ministry of Health, Labour, and Welfare. However, unignorable numbers of family members still feel stigma. New policies are necessary considering the influencing factors.

## Graphical abstract

In 2004, the Japanese government changed the previous Japanese stigmatic term of dementia (“Chiho”) to a present one (“Ninchi‐sho”) a meaning near “neurocognitive disorder.” This study aimed to examine whether the present Japanese term functioned well or not from the viewpoint of families of people with dementia (PWD), and to discover variables influencing their feelings of the term using a structural equation modeling (*SEM*). Overall, the change of the term successfully reduced terminological discomforts in families and the *SEM* found various factors dynamically affecting family members’ feelings, which are useful for making new policies.

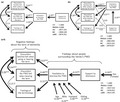

## 1 BACKGROUND

Globally, around 50 million people have dementia, and there are nearly 10 million new cases in 2015 (World Health Organization,). Dementia treatment and care are common worldwide health issues. In 2013, the G8 Dementia Summit declared global action against dementia with an international commitment until 2025 (G8 Dementia Summit declaration,). The declaration called upon all sectors to treat people affected by dementia with dignity and respect and called upon civil society global efforts to reduce stigma, exclusion, and fear. Each country made, and has been running, their own national strategy, cooperatively with other countries.

Reflected in these efforts, social attitudes to people with dementia (PWD) could be changing little by little in some areas. In the United Kingdom, a survey of public attitudes toward PWD showed positive feelings to dementia overall and gender played a role, with younger men having more positive scores than other groups (Cheston et al., 2016). A following study revealed that contact with PWD increases more person‐centered attitudes and suggested that social attitude decreases stigmatizing views (Cheston et al., 2019). In a survey in Japan, nearly 90% of the participants responded that they could make a good relationship with PWD and help them if needed, and analysis showed that information from television and educational classes was associated with such positive attitudes (Aihara et al., 2020).

Regarding self‐stigma in PWD themselves, a qualitative study in the UK showed that the stigma led PWD to hide their diagnosis from others, even close family members (Xanthopoulou & McCabe, 2019). A longitudinal study in the USA found that self‐stigma was associated with poor quality of life outcomes in persons with early‐stage dementia (Burgener et al., 2015). As for families of PWD, a study in Israel showed that caregiver‐stigma increases the caregiver's burden in the case of Alzheimer's disease (Werner et al., 2012). A study in the USA revealed that family caregivers, particularly adult children and female caregivers may experience higher levels of stigma and burden (Kahn et al., 2016). Again in the USA, a qualitative study suggested that shame could underly family stigma which resulted in the family's isolation and delay in access to diagnostic and supportive services for their PWD (Lopez et al., 2020). Thus, comparing social attitudes, the self‐stigma of PWD and their families is quite difficult to change.

Needless to say, the stigma may be tied to there being no remedy for dementia. However, the term “dementia” could also impact PWD and their families. The root of “dementia” is the Latin “demens.” It means “out of one's senses, insane, raving, foolish; distracting, wild, reckless” (Dementia). Therefore, the impression of the term of dementia tends to be negative in many societies. For example, “Chidai” which is the Chinese term of dementia literally means “idiotic or silly (Chi) and dull witted (dai)” (Bedford, 2004). It may exacerbate the stigma associated with individuals even with mild cognitive impairment, part of which could convert into dementia (Dai et al., 2013).

As the translation of “dementia,” Japanese used “Chiho,” the character of which was the same as the Chinese, including discriminating and stigmatic meanings. However, in 2004, the Ministry of Health, Labour, and Welfare (MHLW) in Japan considered the suffering of PWD and that of their families and changed the term to the present one (“Ninchi‐sho”): “Ninchi” and “sho” imply “cognitive” and “disorder,” respectively. The MHLW intended to use the present term instead of the previous term “Chiho,” for the following reasons: it gave insult to PWD; it gave people misunderstanding of PWD as they lost all mental abilities; and it gave people a feeling of threat of dementia, causing a possible obstacle to early interventions including treatable dementia. Before the replacement of the term, the MHLW showed 56.2% of respondents in the national investigation felt discomfort and insult or scorn from the previous term.

Decreasing discrimination and stigma due to dementia is a crucial issue of dementia policy common to each country. As an international interest, it is particularly important to examine if the Japanese policy improved the feelings of PWD and that of their families for their quality of lives. About 15 years have passed since the change of the term so this study aimed to examine whether the replacement functioned well or not from the viewpoint of the family of PWD. More directly, we cross‐sectionally surveyed if the present term has contributed to a decrease in their feelings of discrimination regarding dementia. Furthermore, terms are used in human relations. This study also aimed to analyze the correlations between the families’ feelings of the Japanese present term and their feelings of the people surrounding their PWD, simultaneously examining the relationships with the family members’ and PWD’s attributes.

## 2 MATERIALS AND METHODS

### 2.1 Questionnaire

YK and YM constructed the structure and the draft of items of the questionnaire. After that, the content validity of the questionnaire was discussed and it was completed by a group including the writers of the draft, UT, a clinical psychologist, and an assistant professor having experience of care work and social work.

The questionnaire consisted of 2 categories: feelings of family members about the present Japanese term (Ninchi‐sho); and the family members’ own feelings about the people surrounding the family's PWD. The former category included 12 items, divided into 3 parts: (a) feelings of the terminology of dementia that the family members themselves have (4 items), (b) feelings that family members themselves have when they use the present term (3 items) or hear the term, especially from medical providers (2 items), and (c) the feelings that their PWD has when others use the term (3 items). The latter category included 10 items: the feelings of support by disclosing to surrounding people that their family member had dementia (3 items); hesitation to disclose to surrounding people (7 items).

Participants were asked to answer regarding each of the above items using a rating scale: completely agree; agree a little; neither “agree” nor “disagree”; disagree a little; completely disagree.

### 2.2 Participants

A neurosurgical clinic, a neurological clinic and a psychiatric hospital in the south area of Ibaraki Prefecture cooperated with our study. 196 family members consented to participate in our investigation. Among them, we acquired answers from 155 members (response rate: 79.1%). Data of 153 members were analyzed, except 2 members who did not answer one or two categories of the questions (valid response rate: 78.1%).

The upper part of Table 1 shows the characteristics of the participants (family members) with valid responses. The lower part of Table 1 shows the characteristics of their PWD.

**TABLE 1:** Characteristics of family members (participants) and their person with dementia

	*N (%)*
Family members (Participants)	
Sex	
Male	45 (30.0)
Female	105 (70.0)
N/A	3
Age	
30s	3 (2.0)
40s	17 (11.3)
50s	58 (38.4)
60s	41 (27.2)
70 years of age or older	32 (21.2)
N/A	2
Living with the persons with dementia or not	
Live together	106 (70.7)
Live separately	44 (29.3)
N/A	3
Characteristics of areas	
Downtown area	22 (14.6)
Residential area	96 (63.6)
Rural area	33 (21.9)
N/A	2
Relationships	
Son	29 (19.3)
Daughter	61 (40.7)
Husband	14 (9.3)
Wife	24 (16.0)
Son‐in‐law	2 (1.3)
Daughter‐in law	17 (11.3)
Sibling	2 (1.3)
Grandchild	1 (0.7)
N/A	3
Their person with dementia	
Years after diagnosis	
Less than a year	18 (11.8)
A year or more, less than 5 years	79 (51.6)
5 years or more, less than 10 years	46 (30.1)
10 years or more, less than 15 years	10 (6.5)
Characteristics of living (PWD lives in:)	
Their own home	131 (87.9)
Group home	5 (3.4)
Special nursing home for the aged	3 (2.0)
Long‐term care health facility	3 (2.0)
Others	7 (4.7)
N/A	4
Characteristics of areas	
Downtown area	24 (16.1)
Residential area	89 (59.7)
Rural area	35 (23.5)
Other	1 (0.7)
N/A	4

Note

“Others” of “Characteristics of living” included “living in their child's home” (*n* = 2), “living in a general home for the aged” (*n* = 2), “temporarily staying at a long‐term care facility” (*n* = 3). “Others” of “Characteristics of areas” did not have any detailed description.

Abbreviation: N/A, Not answered.

### 2.3 Procedure

Using a written description, a researcher (YM) explained the study to the family members who were the candidates when they accompanied their PWD to see their geriatric doctor at their clinic/hospital between June 2017 and August 2017. To the family members who consented to participate in our investigation, an anonymous questionnaire on paper was given in the clinics. We asked them to self‐complete it in the clinics, and if that was not possible, to send it to our laboratory by postal mail after it was completed.

### 2.4 Ethics

Ethical approval for this study was granted by the ethics committee of the Human Sciences of the University of Tsukuba. After receiving both a written description and oral explanation of the details of the study, representatives of the three medical institutions signed forms to cooperate with the survey. Both a written description and oral explanation of the details of the study were also given to all candidates accompanying family members with PWD who were outpatients of the institutions. The description included information that this was an anonymous survey and, for this reason, answering the questions proved consent to participate in our study without needing to provide a signature.

### 2.5 Analyses

Descriptive analyses were executed to find the percentage of agreement or disagreement for each item. The ratings of the items were given scores as follows: completely agree (5); agree a little (4); neither “agree” nor “disagree” (3); disagree a little (2); completely disagree (1).

More analysis was done for finding factors or variables that influence the feelings of family members about the present term by TN. First, for examining construct validities of the feelings about the present term and the feelings of the people surrounding PWD, we performed parallel analysis (Horn, 1965) to decide the number of factors we should hypothesize in exploratory factor analysis (EFA). In the parallel analysis, using R 3.6.3 (2018), we first automatically generated 20 random data sets of the same size as the observed data. Based on the data sets, we calculated correlation matrices of the sets and carried out the eigenvalue decomposition. Finally, we compared the observed eigen values to the mean of the simulated eigen values. Then, we performed EFA to disclose relationships between items and factors. In EFA, we transformed the minimum residual solution by promax rotation. Moreover, we did pairwise deletion in the parallel analysis and EFA.

Next, from the results of EFA, we calculated sum scores corresponding to the factors and did the analyses based on Structural Equation Modeling (*SEM*). We constructed models of relationships between the factors and fitted these models to compare indices of the goodness of fit (the comparative fit index (CFI) and Root Mean Square Error of Approximation (RMSEA)) and information criterions (Akaike's Information Criterion (AIC), Bayesian information criterion (BIC) and sample‐size adjusted BIC (saBIC)). In terms of the model fit, we decided the base model indicating the factors’ relationships. Then, we added the family members’ and PWD’s attributes shown in Table 1 to the base model and constructed some models of relationships between the factors and attributes. By comparing RMSEA, CFI, AIC, BIC, and saBIC among these models, we decided a final model of the relationships between variables. In the analyses, we estimated parameters by the maximum likelihood estimation with robust (Huber‐White) standard errors and a scaled test statistic that is (asymptotically) equal to the Yuan‐Bentler test statistic. In the *SEM*, we did listwise deletion.

As we mentioned, we used R 3.6.3 (R Core Team 2018) as the software for the statistical analysis and “psych,” (Revelle “lavaan” (Rosseel, 2012) and “lavaanPlot” (Lishinski,) packages for the above analyses.

## 3 RESULTS

Table 2 shows the family members’ answers to the questions of feelings about the present term. We summarized them as follows: agree (“completely agree” and “agree a little”); neither “agree” nor “disagree”; disagree (“disagree a little” and “completely disagree”). 71.6% agreed with the statement that they felt less discomfort from the present term (Ninchi‐sho) compared with the previous one (Chiho). Only 13.2% of the family members agreed that they felt discrimination when they heard the present term, while 57.0% disagreed. 34.6% of them agreed that they felt discomfort about "Ninchi" (the informal abbreviation of the present term), though 25.2% disagreed and 40.1% could not judge to agree or not. 14.8% agreed that other new terms would give less discomfort than "Ninchi‐sho" (the present term), while 41.2% disagreed. The free descriptions of other possible Japanese terms instead of "Ninchi‐sho" included cognitive dysfunction or disability (*n* = 3); (senile) vascular dysfunction (*n* = 2); (Alzheimer's) brain dysfunction (*n* = 1).

**TABLE 2:** Feelings about the new term

Items	*N* (%)				
	Completely agree	Agree a little	Neither “agree” nor “disagree”	Disagree a little	Completely disagree
Feelings of the terminology of dementia that the family members themselves have					
I think that "Ninchi‐sho" (the present word) gives me less discomfort than "Chiho" (the previous word).	53 (35.8)	53 (35.8)	26 (17.6)	6 (4.1)	10 (6.8)
I think that "Ninchi‐sho" is a discriminatory word.	5 (3.3)	15 (9.9)	45 (29.8)	45 (29.8)	41 (27.2)
I think that "Ninchi" (the abbreviation of the present word) gives me more discomfort than "Ninchi‐sho."	18 (12.2)	33 (22.4)	59 (40.1)	20 (13.6)	17 (11.6)
I think that if there were other new words, they would give me less discomfort than “Ninchi‐sho.”	7 (4.7)	15 (10.1)	65 (43.9)	41 (27.7)	20 (13.5)
Feelings that family members themselves have when they use or hear the term					
I feel discomfort to use "Ninchi‐sho" to a family member with dementia.	22 (14.5)	30 (19.7)	30 (19.7)	45 (29.6)	25 (16.4)
I feel discomfort to use "Ninchi‐sho" to other family members or relatives.	10 (6.5)	27 (17.6)	23 (15.0)	58 (37.9)	35 (22.9)
I feel discomfort to use "Ninchi‐sho" to my friends or neighbors.	16 (10.5)	28 (18.3)	28 (18.3)	53 (34.6)	28 (18.3)
I feel discomfort for "Ninchi‐sho" to be used by medical providers including doctors.	4 (2.6)	8 (5.3)	24 (15.9)	57 (37.7)	58 (38.4)
I recognize the seriousness of the disease again when medical providers, including doctors, use “Ninchi‐sho."	12 (7.9)	38 (25.2)	39 (25.8)	38 (25.2)	24 (15.9)
Feelings that their family member with dementia has when others use the term					
I think my family member with dementia feels discomfort when other family members or my relatives use "Ninchi‐sho."	19 (12.5)	36 (23.7)	46 (30.3)	34 (22.4)	17 (11.2)
I think my family member with dementia feels discomfort when their friends or neighbors use "Ninchi‐sho."	22 (14.5)	39 (25.7)	44 (28.9)	32 (21.1)	15 (9.9)
I think my family member with dementia feels discomfort when medical providers, including doctors, use "Ninchi‐sho."	17 (11.1)	18 (11.8)	44 (28.8)	43 (28.1)	31 (20.3)

Note

Ninchi‐sho = the present Japanese term of dementia; Chiho = the previous term; Ninchi = informal abbreviation of the present term.

Regarding feelings of discomfort when family members used the present term, on the whole, the agreement was less than disagreement. However, relatively, the rate of use to PWD in their family (34.2%) was higher than others: other family members or relatives (24.1%), friends or neighbors (28.8%). As for the medical providers’ use of "Ninchi‐sho," family members hardly worried about the use itself (7.9%). Compared with the feelings, more members agreed that they felt the seriousness of the disease again by hearing "Ninchi‐sho" in medical situations (33.1%).

Considering discomfort which the family thought their PWD had, agreement was more than disagreement: the family members or their relatives (36.2%); their friends or neighbors (40.2%). However, 22.9% agreed that they felt the seriousness of the disease when doctors or specialists used “Ninchi‐sho," while 48.4% disagreed.

Table 3 shows the family members’ answers to feelings about the people surrounding the PWD. For the first two questions regarding support or help from their friends or neighbors if they revealed their family member's dementia, about 60 percent of them agreed, while those who disagreed were less than about 10 percent. Regarding the third question, 79.5% expected that more appropriate treatments would be provided and that their family members would have some improvements if they received a diagnosis.

**TABLE 3:** Feelings of surrounding people's attitude and relationships because of dementia

Items	*N* (%)				
	Completely agree	Agree a little	Neither “agree” nor “disagree”	Disagree a little	Completely disagree
Support by disclosure					
I think I will get more support and help from my friends or neighbors if I tell them that my family member has dementia.	37 (24.5)	60 (39.7)	37 (24.5)	9 (6.0)	8 (5.3)
I think my friends or neighbors will warmly help watch over my family member with dementia, if I tell them that he/she has dementia.	25 (16.7)	59 (39.3)	61 (40.7)	2 (1.3)	3 (2.0)
I think my family member with dementia would be provided more appropriate treatments and have some improvements if the diagnosis is done.	69 (45.7)	51 (33.8)	18 (11.9)	7 (4.6)	6 (4.0)
Hesitation to disclose the PWD					
I think the relationship between me and my friends or neighbors will get worse if I tell them that my family member has dementia.	0 (0.0)	8 (5.3)	35 (23.0)	59 (38.8)	50 (32.9)
I think it will bother my friends or neighbors (that is, make them worry) if I tell them that my family member has dementia.	15 (9.9)	42 (27.6)	36 (23.7)	40 (26.3)	19 (12.5)
I think I will disgrace my family including the member with dementia in public if I tell my friends or neighbors that he/she has dementia.	2 (1.3)	15 (9.9)	25 (16.4)	49 (32.2)	61 (40.1)
I think my friends or neighbors will give my family member with dementia discriminatory looks if I tell them that he/she has dementia.	5 (3.3)	27 (17.9)	34 (22.5)	52 (34.4)	33 (21.9)
I think my family member with dementia will be looked down on if I tell friends or neighbors that he/she has dementia.	9 (6.0)	25 (16.6)	38 (25.2)	46 (30.5)	33 (21.9)
I think few friends or neighbors of mine have correct knowledge about dementia.	25 (16.6)	46 (30.5)	55 (36.4)	20 (13.2)	5 (3.3)
I was afraid that my family member would be diagnosed as having dementia, so I hesitated to take them to a doctor.	5 (3.3)	10 (6.7)	21 (14.0)	45 (30.0)	69 (46.0)

Considering questions about the feelings of the family members to hesitate to disclose their PWD to surrounding people, very few family members (5.3%) agreed that the relationship between them and their friends or neighbors would get worse if they revealed the PWD. However, 37.5% of them agreed that it would bother their friends or neighbors in some way. Only 11.2% agreed that their PWD would be disgraced in public if they told their friends or neighbors about the dementia. 22.6% of them agreed that the family member would be looked down on, while 52.4% disagreed. In medical settings, only 10.0% of them hesitated to take family members to a doctor because of a concern about their family member being diagnosed. However, considering correct knowledge about dementia, 47.1% of them agreed that their friends or neighbors did not have such knowledge.

Table 4 shows the result of EFA regarding the feelings of family members about the present term. Because the result of parallel analysis suggested that there might be two or three factors, we fitted two and three factor EFA models to the data and compared the goodness of fit indices (Tucker Lewis Index (TLI) of factoring reliability and RMSEA). These indices indicated that the three‐factor model fitted better: TLI (0.92); RMSEA (0.09). Therefore, we used the three‐factor model. The first factor essentially consists of the items regarding family's discomfort when they use or hear the present term in the second group of items in Table 2, except an item of discriminatory perception of the term included in the first group in Table 2. The second factor included the items in relation to feelings of family members regarding PWD's discomfort when they hear the present term: the third group of items in Table 2. The last factor basically comprised feelings of the terminology of dementia, corresponding to the first group of items in Table 2, while only the item of discriminatory perception of the term moved into the first factor, as above mentioned.

**TABLE 4:** Explanatory factor analysis of discomfort from terms relating to dementia

Items	Factor 1	Factor 2	Factor 3
I feel discomfort to use "Ninchi‐sho" to other family members or relatives.	**1.00**	−0.12	−0.05
I feel discomfort to use "Ninchi‐sho" to my friends or neighbors.	0.**88**	−0.05	−0.03
I feel discomfort for "Ninchi‐sho" to be used by medical providers including doctors.	0.**86**	−0.05	−0.16
I feel discomfort to use "Ninchi‐sho" to a family member with dementia.	0.**63**	0.15	0.04
I think that "Ninchi‐sho" is like a discriminatory word.	0.**54**	0.25	0.03
I recognize the seriousness of the disease again when medical providers, including doctors, use “Ninchi‐sho."	0.**34**	0.11	0.09
I think my family member with dementia feels discomfort when other family members or my relatives use "Ninchi‐sho."	−0.11	0.**99**	0.06
I think my family member with dementia feels discomfort when their friends or neighbors use "Ninchi‐sho."	−0.03	0.**94**	0.05
I think my family member with dementia feels discomfort when medical providers, including doctors, use "Ninchi‐sho."	0.12	0.**75**	−0.15
I think that "Ninchi‐sho" gives me less discomfort than "Chiho."	−0.14	−0.06	0.**81**
I think that "Ninchi" gives me more discomfort than "Ninchi‐sho"	−0.04	0.08	0.**58**
I think that if there were other new words, they would give me less discomfort than “Ninchi‐sho.”	0.**39**	−0.10	0.**48**
Percentages of explained variance (%)	48	35	17
Correlation coefficients		0.65	0.47
			0.36

Note

The numerals after promax rotation are shown. The ones above 0.30 factor loading are indicated in boldface. Ninchi‐sho = the present Japanese term of dementia; Chiho = the previous term; Ninchi = informal abbreviation of the present term.

Table 5 shows the result of factor analysis of feelings of the family members to surrounding people. In the analysis, we hypothesized two factors based on the result of parallel analysis. The fitness of the model was satisfactory: TLI (0.94); RMSEA (0.07). The first factor and the second factor included items relating to hesitation of the family members to disclose their PWD to surrounding people, and items regarding support by disclosure, respectively, corresponding to the second group and the first group of items in Table 3.

**TABLE 5:** Explanatory factor analysis of feelings of others' attitude and relationships

Items	Factor 1	Factor 2
I think my friends or neighbors will give my family member with dementia discriminatory looks if I tell them that he/she has dementia.	0.**87**	0.07
I think it will bother my friends or neighbors (that is, make them worry) if I tell them that my family member has dementia.	0.**77**	0.12
I think my family member with dementia will be looked down on if I tell friends or neighbors that he/she has dementia.	0.**77**	0.03
I think I will disgrace my family including the member with dementia in public if I tell my friends or neighbors that he/she has dementia.	0.**75**	−0.10
I think the relationship between me and my friends or neighbors will get worse if I tell them that my family member has dementia.	0.**68**	−0.09
I was afraid that my family member would be diagnosed as having dementia, so I hesitated to take them to a doctor.	0.**60**	0.08
I think few friends or neighbors of mine have correct knowledge about dementia.	0.**38**	−0.23
I think my friends or neighbors will warmly help watch over my family member with dementia, if I tell them that he/she has dementia.	−0.09	0.**87**
I think I will get more support and help from my friends or neighbors if I tell them that my family member has dementia.	0.05	0.**54**
I think my family member with dementia would be provided more appropriate treatments and have some improvements if the diagnosis is done.	0.04	0.**35**
Percentages of explained variance (%)	73	27
Correlation coefficients		−0.43

Note

The numerals after promax rotation are shown. The ones above 0.30 factor loading are indicated in boldface.

**FIGURE 1:**
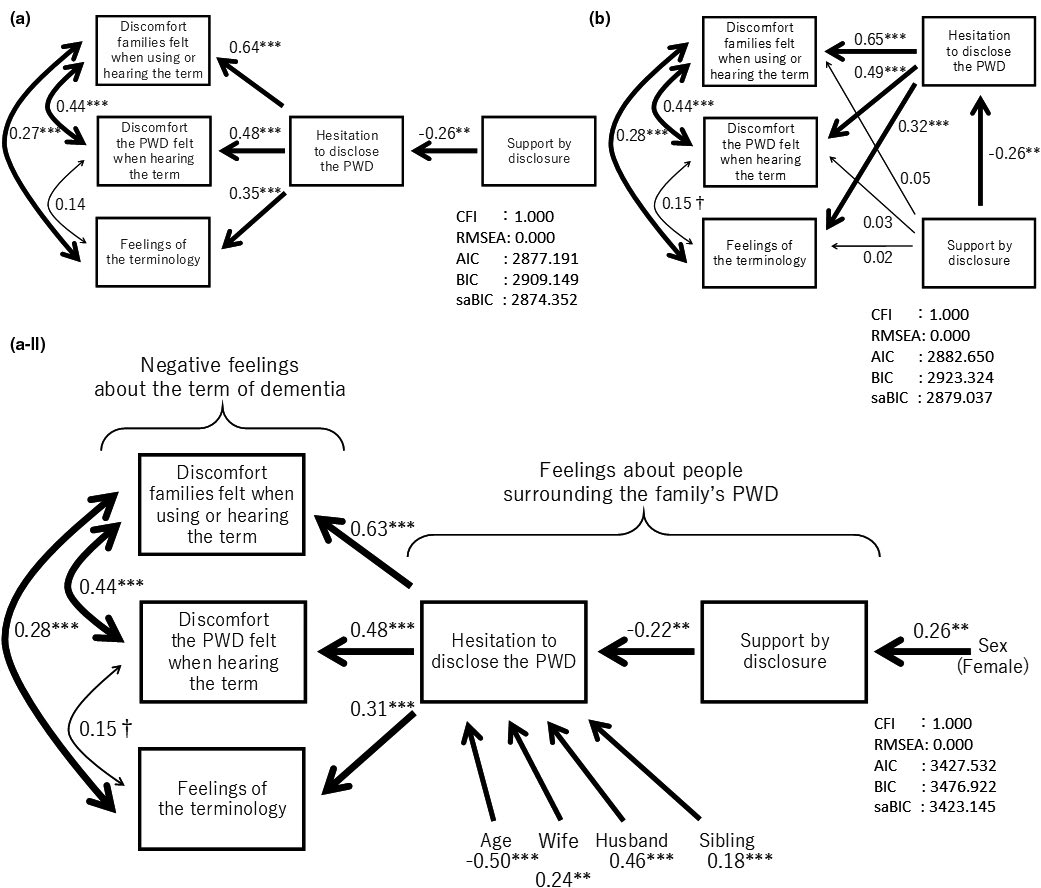
Results of structural equation modeling for the overall structure of feelings about the new term of dementia with other factors and attributes (*N* = 135). *Note*. All coefficients were standardized. For simplicity, error terms were not shown. Bold lines indicate statistically significant paths. CFI = Comparative Fit Index; RMSEA, Root Mean Square Error of Approximation; AIC, Akaike's Information Criterion; BIC, Bayesian Information Criterion; saBIC, Sample‐size Adjusted Bayesian Information Criterion. †*p* < .10, **p <.01, **p <.001

Models of the relationship of extracted factors from two factor analyses were constructed. In all combinations of the factors we considered, Model A and Model B in Figure 1 were the first and second best from the indices of the fitness calculated by the *SEM*. (A) was the model of the family members’ feelings of hesitation to disclose the dementia directly influencing the feelings of the present term and the feelings of family members supported from surrounding people by disclosure of their PWD which indirectly influenced the feelings of the present term, mediated by the feelings of hesitation to disclose. (B) was the model of both the two feelings of the family members to surrounding people directly influencing the feelings of the present term. The two models indicated enough fitness (CFI = 1.000 and RMSEA = 0.000). However, in terms of information criterions (AIC, BIC, and saBIC), Model A showed a better fit to the data than Model B. Additionally, in Model B, the feelings of family members supported from surrounding people by disclosure of their PWD had little direct (nonsignificant) impact on the feelings of the present term.

Next, based on Model A, more models were created by combining the family members’ and PWD’s attributes. Among the models, model A‐II in Figure 1 was that with the highest fitness, considering the above indicators. In this model, the attributes of the family members such as a younger age, wives, husbands, and siblings significantly influenced the feelings of hesitation to disclosure the dementia. Moreover, sex (female) significantly impacted the feeling of family members supported from surrounding people by disclosure.

## 4 DISCUSSION

Originally, “dementia” was translated as “Chiho,”, the previous Japanese term by Shuzo Kure who was a professor of psychiatry in Tokyo Imperial University in 1908. However, it seems that the general usage of “Chiho” in Japanese society was confused for decades. In 1955, the definition in “Koujien,” a popular Japanese dictionary at that time included “stupid” or “idiot” (Ministry of Health, Labour, & Welfare). In our findings, about 70% of the participants felt less discomfort from "Ninchi‐sho" (the present term) compared to “Chiho” (the previous term). Moreover, only about 13.0% of the participants thought “Ninchi‐sho” was discriminatory. These results suggested that overall, the policy for changing the term was successful, considering the result of feelings of the previous term in the survey done by the Japanese MHLW before the replacement of the term.

However, it is noteworthy that about 35% of the participants felt “Ninchi,” the informal abbreviation of the present term gave them more discomfort than "Ninchi‐sho." Some Japanese medical and social care specialists often use the abbreviation as an indirect wording of "Ninchi‐sho," as they consider the negative impact the full term gives. The result suggested that professionals should consider that a certain number of family members might also feel discomfort from the abbreviation. Some family members may feel that the abbreviation of the diagnosis is slang and also contains a hidden discriminatory meaning, regardless of the users’ intention. Moreover, we should not ignore that a certain percent of the participants still felt discomfort when they use even the present term. We also cannot disregard that a considerable number of families thought their PWD felt discomfort from the term. Such people may feel discomfort about the term although they recognize it is not discriminatory inside their own mind. We should make strategies in order to decrease these hidden negative feelings.

For finding clues, we examined the relationship of the feelings of the term with feelings of family members regarding people surrounding PWD, and the attributes of the families and the PWD, using *SEM*. Before the analysis of *SEM*, EFA was performed to examine construct validities of the feelings about the present term and the feelings of the people surrounding PWD. As a result, the extracted factors approximately matched with assumed categories of constructs, which indicated that the construct validities were confirmed. Using these constructs, the result from analysis of *SEM* showed that the feelings of support from others could not directly alleviate the negative feelings of the term. Instead, the feelings of support indirectly influenced the feelings through hesitation to disclose the dementia, that is, negative feelings to surrounding attitudes.

From the result, decreasing hesitation to disclose the dementia could be of primary importance in order to change the negative feelings of the term. As a clue to develop the strategy, half of the family members felt that their friends or neighbors did not have correct knowledge of dementia, which was more negative than the answers to other questions. Currently, most Japanese people have recognized the term and existence of dementia, but they are extremely interested in prevention rather than care. Fewer people may know the symptoms and care of dementia and understand the necessity of creating a “dementia friendly community.” Family members could have some supportive people for their PWD, but half of the members felt that supporters lacked knowledge. If family members asked the people to help, they could rather have more a burden to support the people; they needed to tell supporters how to care for the PWD. This may be a Japanese characteristic but most Japanese automatically think such interactions will also bother the people because of taking more time. In this study, about 40% of the family members thought it would bother their friends if they revealed the dementia which their family member had. That result might be linked to their feelings of the lack of knowledge of the surrounding people. If so, municipal governments should drastically proceed education for informal dementia care in each community. Moreover, younger family members, wives, husbands, and siblings of the PWD tend to have more hesitation to disclose the dementia. Younger agers’ hesitation was in accordance with previous studies of stigma from dementia (Kahn et al., 2016). Moreover, a study with general people as the subjects suggested that people have a trend to perceive stigma of dementia in their “acquaintance community” (Gao et al., 2020). Relating to this study, our result in the *SEM* may show that wives, husbands, and siblings who knew neighbors of PWD more than other relatives, tended to hide the dementia of the PWD by being scared of “loss of face” (Woo, 2017). Such hesitation could lead them to be isolated in their community. For example, to cope with the problem, enhancement of accessible peer support groups and having facilitators in the community where they live are important.

From the result of *SEM*, it is also important to focus on the factor “support by disclosure.” For more improvement of stigma from the terminology of dementia, making more supportive attitudes from others and structuring communities friendly to PWD are essential. These could break the families’ negative feelings to surrounding people and gradually change stigma by the terminology. It is noteworthy that female family members had more feelings of support by disclosure, which corresponded to the previous studies done with general subjects (Stites et al., 2018; Wadley & Haley, 2001). As a strategy, by targeting females, if more cases of successful support from surrounding people are well‐known in communities, more family members could trustingly ask others for support with their PWD, without hesitation. Eventually, such efforts may lead to a greater decrease in discomfort and stigma from the terminology.

As a conclusion, this study found that most Japanese family members accepted the present Japanese term which the government created with the intention to reduce the feelings of discrimination. On the whole, it was suggested that the present term successfully reduced discomfort in families, compared with the result of the previous term surveyed by the Japanese MHLW. However, we should pay attention to the fact that even the present term still gave a non‐negligible number of participants discomfort and we should consider other strategies to make more family members feel less discomfort and stigma. The result from *SEM* gave clues: decrease of hesitation to disclose dementia and increase of success of support, linked to sex and the relative's characteristics of the family members.

However, we should mention limitations of this study. The data were sampled in three hospitals in a local area in Japan. For the general conclusion, a more global, nation‐wide survey will be necessary. Validity of the questionnaires should be examined more using a larger sample. We should also analyze more variables, such as the severity of dementia, which we could not do in this study. Furthermore, we asked the family members about the discomfort that PWD felt from the terminology. The result showed that the family members thought PWD felt more discomfort compared with the family members. However, we could not reveal the precise degree to which PWD felt from the possibility of bias; less agreement of discomfort they felt when medical providers used the present term than disagreement. Therefore, it is important to study the present Japanese term of dementia from the viewpoint of PWD themselves.

## CONFLICT OF INTEREST

None declared.

## AUTHOR CONTRIBUTION

Katsuo Yamanaka involved in design of the study, development of the questionnaire, design of the analyses, interpretation of the results, and writing of the manuscript (except the section of multivariate analyses) as the leader of this project. Naoya Todo involved in multivariate analyses and the writing of that section of the manuscript. Mutsumi Yoshizawa involved in development of the questionnaire, investigation, and descriptive analysis. Tatsuji Uchida involved in development of the questionnaire and interpretation of the results. All authors involved in writing of the final paper.

### Peer Review

The peer review history for this article is available at https://publons.com/publon/10.1002/brb3.2012.

## Data Availability

Data available within the article or its supplementary materials.
